# Feasibility of combining two individualized lung recruitment maneuvers at birth for very low gestational age infants: a retrospective cohort study

**DOI:** 10.1186/s12887-020-02055-3

**Published:** 2020-04-01

**Authors:** Zalfa Kanaan, Coralie Bloch-Queyrat, Marouane Boubaya, Vincent Lévy, Pascal Bolot, Paul Waszak

**Affiliations:** 1 Service de Réanimation Néonatale et Néonatalogie, Hôpital Delafontaine, 2 rue Dr Delafontaine, 93205 Saint-Denis, France; 2 Unité de Recherche Clinique, Groupe Hospitalier Paris Seine Saint-Denis, APHP, Bobigny, France

**Keywords:** Neonatal resuscitation, Lung recruitment, Dynamic PEEP, Sustained inflation, Bronchopulmonary dysplasia

## Abstract

**Background:**

Lung recruitment at birth has been advocated as an effective method of improving the respiratory transition at birth. Sustained inflations (SI) and dynamic positive end-expiratory pressure (PEEP) were assessed in clinical and animal studies to define the optimal level. Our working hypothesis was that very low gestational age infants (VLGAI) < 32 weeks’ gestation require an individualized lung recruitment based on combining both manoeuvers.

**Methods:**

Between 2014 and 2016, 91 and 72 inborn VLGAI, requiring a respiratory support beyond a continuous positive airway pressure (CPAP) = 5 cmH2O, were enrolled before and after introducing these manoeuvers based on progressive increase in SI up to 15 s, with simultaneous gradual increase in PEEP up to 15 cmH2O, according to the cardiorespiratory response. Retrospective comparisons of the incidence of mechanical ventilation (MV) < 72 h of life, short-term and before discharge morbidity were then performed.

**Results:**

Among extremely low gestational age infants (ELGAI) < 29 weeks’ gestation, the following outcomes decreased significantly: intubation (90 to 55%) and surfactant administration (54 to 12%) in the delivery room, MV (92 to 71%) and its mean duration < 72 h of life (45 h to 13 h), administration of a 2nd dose of surfactant (35 to 12%) and postnatal corticosteroids (52 to 19%), and the rate of bronchopulmonary dysplasia (23 to 5%). Among VLGAI, all of these results were also significant. Neonatal mortality and morbidity were not different.

**Conclusions:**

In our setting, combining two individualized lung recruitment maneuvers at birth was feasible and may be beneficial on short-term and before discharge pulmonary outcomes. A randomized controlled trial is needed to confirm these results.

## Background

Resuscitating premature infants at birth aims to stimulate spontaneous breathing and establish an optimal functional residual capacity without harming the lungs [1]. To this end, lung recruitment maneuvers have been under investigation over the past two decades [2–6]. To date, despite numerous studies, no clear definition of the optimal inflation duration, nor of positive end-expiratory pressure (PEEP) optimal level exists. According to current knowledge on the respiratory transition at birth [7], sustained inflation (SI) aerates the fluid-filled lungs homogeneously during the initial fetal-neonatal transition phase, and thereafter, adequate PEEP helps to maintain the fluid in the interstitial tissue.

Current international guidelines recommend starting resuscitation of premature infants with intermittent positive pressure ventilation, using a PEEP of 5 cmH2O with the first insufflations of 2–3 s (but not greater than 5 s) [8, 9]. However, these measures are probably not sufficient for recruiting the optimal functional residual capacity.

Lung recruitment (LR) at birth using SI of 15–20 s was assessed in numerous randomized controlled trials (RCTs) summarized in 2 recent meta-analyses [10, 11]. Despite encouraging results such as a reduced need for intubation, no benefit was found for reduction of mortality or bronchopulmonary dysplasia (BPD). However, studies in the premature lamb have shown better oxygenation, lung mechanics, and end-expiratory global lung volume after a dynamic PEEP strategy based on a step-by-step PEEP increase far beyond 5 cmH2O compared with a nonoptimized SI strategy [4, 6]. However, none of these differences were observed when the dynamic PEEP strategy was compared with a SI strategy optimized by real time electrical impedance tomography for the duration of the inflation [5]. We hypothesized that individualized LR maneuvers combining dynamic PEEP and gradually extended SI, as needed, would lower the incidence of mechanical ventilation (MV) in the first 72 h, without increasing neonatal complications. To this end, we retrospectively compared short-term and before discharge outcomes, before and after introducing LR maneuvers in very low gestational age infants (VLGAI) < 32 weeks of gestation and especially in the subgroup of extremely low gestational age infants (ELGAI) < 29 weeks of gestation, with higher morbidity and mortality rates.

## Methods

### Population

Our tertiary level hospital covers the Saint-Denis area in the north of Paris, France, with a rate of ~ 4500 deliveries/year. Inborn VLGAI requiring pulmonary resuscitation born from July 2014 to June 2015 (*n* = 91) and for all of 2016 (*n* = 72) were included in this retrospective cohort study. A 6-month gap between both periods was needed to introduce LR maneuvers to the NICU team. Data were collected from patients’ medical records and reports.

A total of 242 VLGAI were managed during these periods (119 during 2014/2015 and 123 during 2016). We excluded infants transferred from other hospitals (*n* = 12) and (*n* = 14), infants suffering from major congenital anomalies (*n* = 3) and (n = 3), and newborns requiring no respiratory support beyond a CPAP value of 5 cmH2O (*n* = 13) and (*n* = 10) in 2014/2015 and 2016, respectively. Respiratory support was started based on currently used international guidelines [8, 9]. The adherence to the new protocol was judged on the written words describing the resuscitation in the DR. During the second period, 24/96 (25%) pulmonary resuscitations did not follow the LR algorithm and were excluded from the analysis.

Data collection was approved by the local area ethics committee on human research (Comité Local d’Ethique d’Avicenne belonging to Groupe Hospitalier Paris-Seine-Saint-Denis) which allowed a waiver of informed consent for this retrospective study (Protocol No. CLEA-2017-031).

### Intervention (Fig. 1)

The intervention was the introduction of the LR strategy. In the 2014/2015 period, the respiratory assistance of VLGAI was conducted according to international recommendations (PEEP of 5 cm H2O, inflation duration of 2–3 s, peak inspiratory pressure of 20–25 cmH2O) using a face mask with a T-piece resuscitator (Neopuff, Fisher & Paykel, Auckland, New-Zealand), followed by intubation if the baby remained apneic or cyanotic with a pulse oxygen saturation (SpO2) < 75% at 5 min of life whatever the fraction of inspired oxygen (FiO2), or with a heart rate (HR) < 100/min or an FiO2 > 0.4 at 10 min of life in order to achieve an SpO2 > 85%. During 2016, respiratory support was changed to gradual increase in inflations duration (from 3 to 5, to 10, then 15 s), as needed, and performed with a simultaneous increase in PEEP every 1 to 2 SI from 5 to 8, 10, 12, then 15 cmH2O, according to the clinical response, which included persistent bradycardia, apnea or gasping. A gradual decrease in PEEP was performed when FiO2 reached 0.4, while SIs were stopped when regular spontaneous breathing was observed. In case of severe asphyxia, emergency intubation with or without chest compressions did not cancel the LR strategy. During both periods, the resuscitation team included at least a neonatologist, a pediatric resident, and a midwife. The team member at the head of each newborn kept track of the titrating of SIs by counting aloud each duration, and announcing the PEEP level set at the PEEP cap located on the T-piece. The PIP level which was not modified with the PEEP level, was eventually increased by turning the ad-hoc knob by the team member closest to it.

**Fig. 1:**
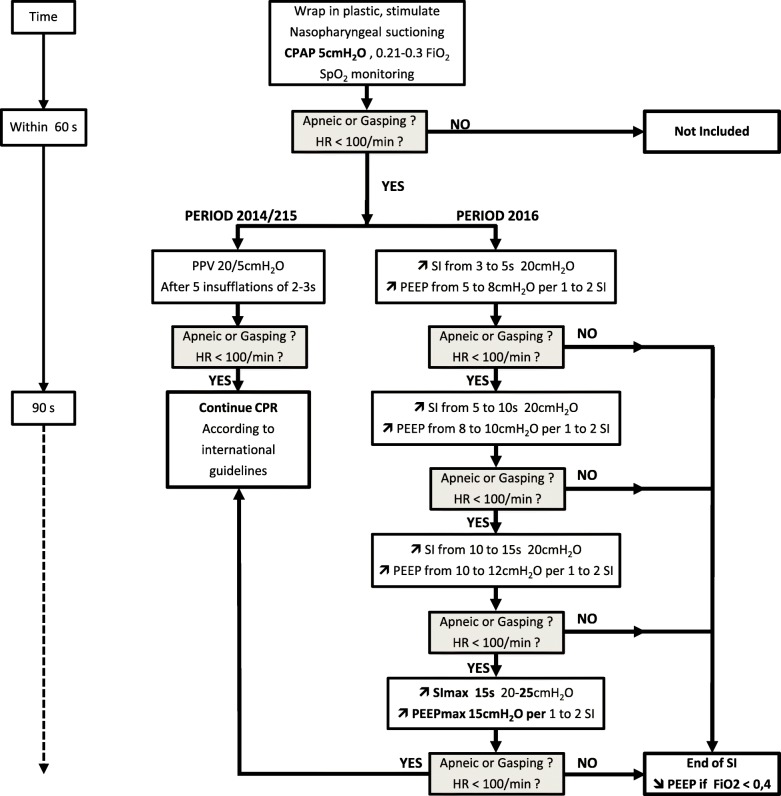
Resuscitation algorithm in the delivery room. CPAP: continuous positive airway pressure; FiO2: fraction of inspired oxygen; SpO2: oxygen saturation; HR: heart rate; PPV: positive pressure ventilation; SI: sustained inflation; PEEP: positive end-expiratory pressure; CPR: cardiopulmonary resuscitation

### Monitoring in the DR

Postnatal hypothermia was prevented by using radiant heaters and warmed blankets. After drying, extremely low gestational age infants (ELGAIs) < 29 weeks were immediately wrapped in a sterile, transparent plastic bag. The head was covered by a warmed cap. Temperature was monitored via a cutaneous sensor. HR was measured by stethoscope and then monitored by an SpO2 sensor (Massimo™) placed at the right hand.

### Transport to the neonatal intensive care unit (NICU)

A preheated neonatal transport incubator was used for the transfer to the NICU. During both periods, infants were ventilated (invasively or not) using a Babylog 8000 (Dräger, Lübeck, Germany). Continuous positive airway pressure (CPAP) or noninvasive ventilation (NIV) was provided during the transfer by using nasal cannula (Neotech RAM Cannula™, Valencia, CA, USA).

### Co-interventions

#### Obstetric management

Some changes in obstetric treatment were introduced in October 2016: antibiotic therapy duration after preterm premature rupture of membrane was shortened from 5 to 3 days [12]; and magnesium sulfate was infused to pregnant women prior to the preterm birth of infants < 33 weeks’ gestation [13].

#### NICU management

Neonatal management was as follows:

- Intubation criteria did not change: (i) in the DR, they remained as stated above; (ii) in the NICU, the criteria were an FiO2 > 0.4, frequent (> 6 occurrences during 6 h) or severe apneas (> 1 per 6 h requiring bag-mask ventilation).
- CPAP or NIV was maintained using the InfantFlow® biphasic nCPAP device (SEBAC, Gennevilliers, France). The synchronized mode was used in case of hypercapnia or moderate apnea despite caffeine treatment.
- During 2016, permissive hypercapnia was introduced with a maximal tolerated pCO2 limit of 65 mmHg (vs 55 mmHg during 2014/2015).
- Oxygen level was adjusted to obtain an SpO2 target range of 87–93% during 2016 instead of 85–95% during 2014/2015.
- During 2016, the maintenance dose of caffeine (5 mg/kg/day) was doubled in case of moderate apnea.
- During the second period, the first surfactant administration (200 mg/kg of Curosurf, Chiesi, Parma, Italy) was indicated when FiO2 exceeded 0.25 and 0.30 in ELGAIs and VLGAIs respectively, instead of 0.4 during the first period, whatever the gestational age (GA). During both periods, a second dose (100 mg/kg) was administered when FiO2 exceeded 0.4. If the infant was not intubated, the intubation-surfactant-extubation (INSURE) procedure was performed, and considered successful (without MV) if the extubation was carried out less than 1 hour later.
- Postnatal corticosteroid therapy criteria remained the same for premature infants more than 21 days old: persistent need for MV with FiO2 exceeding 0.4, or after failure of extubation.
- During 2016, early-rescue high frequency oscillation (HFO) ventilation was introduced in case of INSURE failure or an early (< 72 h of life) need for invasive PPV, while volume guarantee ventilation was introduced to accelerate the weaning process. A change from a systematic sedation-analgesia toward an individualized approach based on the EDIN scale [14] was also performed in invasively ventilated infants.
- No major changes in the nutrition protocol occurred, except for probiotics (*Lactobacillus casei* and *Lactobacillus rhamnosus*) introduced in April 2016.

### Primary and secondary outcomes

The primary outcome was the incidence of MV in the first 72 h. Secondary outcomes were the rate of intubation in the DR; Apgar score at 5 min; surfactant use; ventilator days; postnatal corticosteroids; mortality and morbidity including physiologic BPD [12]; pneumothorax; intraventricular hemorrhage (IVH) > grade 2 [13]; periventricular leukomalacia [14]; treated patent ductus arteriosus (PDA); necrotizing enterocolitis (NEC) ≥ stage 2a [15]; and retinopathy of prematurity (ROP) > stage 2 [16].

### Statistical analysis

Data are summarized as median and interquartile range for quantitative data and as count and percentage for categorical data. Statistical analyses were performed using chi-squared test or Fisher’s exact test for categorical variables and the t-test or Mann-Whitney U-test for continuous variables. All outcomes with *p* < 0.10 in univariate analysis were analyzed with multiple logistic regression. Adjustments were made for confounding, such as sex, birth weight, GA, mode of delivery and low Apgar score (< 5) at 1 min. Similar analyses were performed for the infants of GA between 29 and 31 weeks and ELGAI subgroup. No adjustment of *P* values was performed to account for multiple comparisons because subgroups analyses are considered exploratory. All tests were two-sided at a 0.05 significance level. Analyses were carried out using R statistical software version 3.3.2.

## Results

### Comparability of study groups (Table 1)

Although both periods were similar regarding all baseline population characteristics, the cesarean section rate increased from 58 to 74% without reaching significance (*p* = 0.06).

**Table 1:** Very Low Gestational Age Infants < 32 weeks GA Population Characteristics

	Period 1 (2014/2015) *N* = 91	Period 2 (2016) *N* = 72
Female, n (%)	41 (45)	30 (42)
Gestational age, weeks (median) [1st Qu; 3rd Qu]	28 [27;30]	28 [27;30]
Birth weight, g (median) [1st Qu; 3rd Qu]	1030 [850;1356]	1028 [879;1344]
IUGR, n (%)	21 (23)	17 (24)
Cesarean Section, n (%)	53 (58)	53 (74)
Premature birth causes, n (%)		
- Chorioamnionitis	28 (31)	14 (19)
- Pre-eclampsia + abnormal FHR	38 (42)	41 (57)
- Other	12 (13)	9 (13)
- Idiopathic	13 (14)	8 (11)
Antenatal corticosteroids, n (%):		
- None	15 (17)	8 (11)
- One dose	21 (23)	12 (17)
- Two doses	55 (60)	52 (72)

*GA* Gestational age, *IUGR* Intrauterine growth restriction (< 3rd percentile), *FHR* Fetal heart rate; No significant difference between period 1 and 2

### Intervention

As stated above, in 2016, 10 newborns requiring no respiratory support beyond a CPAP value of 5 cmH2O were excluded from the study, as were also excluded 24/96 (25%) newborns who did not benefited from the LR algorithm. That is, without any written word on increased inflation time > 3 s or PEEP value > 5 cmH2O despite a low Apgar score. Data concerning the LR maneuvers in 2016 were available for 66/72 (92%) patients: 20 (30%) infants required inflations with a peak inspiratory pressure of 20 cmH2O but without any recruitment maneuvers beyond five 3-s-inflations, 13 (20%) required an SI = 5 s with a PEEP = 8 cmH2O, 24 (36%) required an SI = 10 s with a PEEP = 10–12 cmH2O, while only 9 (14%) required an SI = 15 s with a PEEP = 15 cmH2O. Without reaching significance (*p* = 0.58 and *p* = 0.23, respectively) the median maximal SI and PEEP were higher in the ELGAI subgroup (10 s and 10 cmH2O) than in the 29- to 31-week subgroup (5 s and 8 cmH2O) (Table 2).

**Table 2:** Delivery Room Management Data

	VLGAI		29- to 31-wk stratum		ELGAI	
	Period 1 N = 91	Period 2 N = 72	Period 1 *N* = 43	Period 2 *N* = 30	Period 1 *N* = 48	Period 2 *N* = 42
SImax, s (median) [1st Qu; 3rd Qu]	2 [2–2]	5 [3–10]	2 [2–2]	5 [3–10]	2 [2–2]	10 [5–10]
PEEPmax, cmH2O (median) [1st Qu; 3rd Qu]	5 [5–5]	8 [5–10]	5 [5–5]	8 [5–10]	5 [5–5]	10 [7–11]
5 min Apgar score < 5, n (%)	7 (8)	1 (1)	3 (7)	0 (0)	4 (8)	1 (2)
10 min Apgar score < 5, n (%)	1 (1)	1 (1)	0 (0)	0 (0)	1 (2)	1 (2)
Intubation in the delivery room, n (%)	68 (75)	26 (36)*##	25 (58)	7 (23) *##	43 (90)	19 (55)*##
Adrenaline administration, n (%)	7 (8)	1 (1)	4 (9)	0 (0)	3 (6)	1 (2)
Chest compressions, n (%)	5 (6)	1 (1)	5 (12)	0 (0)	0 (0)	1 (2)
Surfactant administration, n (%)	29 (32)	9 (13) *##	3 (7)	4 (13)	26 (54)	5 (12) *##

VLGAI: very low gestational age infants < 32 weeks; ELGAI: extremely low gestational age infants < 29 weeks; SImax: maximal sustained inflation; PEEPmax: maximal positive end-expiratory pressure; *##: p and adjusted *p* < 0.01

### DR management data (Table 2)

The rate of the Apgar score < 5 at 5 min was divided by 8 in VLGAI and by 4 in ELGAI, while no Apgar score < 5 at 5 min was observed in the 29- to 31-week stratum, but without reaching significance. A similar fall in the rate of adrenaline administration in the DR was observed without reaching significance. The rate of chest compressions was also not significantly divided by 6 in VLGAI, while no more chest compressions were practiced in the 29- to 31-week subgroup. Moreover, the rate of intubation in the DR significantly decreased by 52% in VLGAI (*p* < 0.01), and specifically by 60% in the 29- to 31-week subgroup (*p* < 0.01), and by 39% in the ELGAI subgroup (*p* < 0.01). Exogenous surfactant administration in the DR decreased significantly by 59% in VLGAI (*p* < 0.01), and especially by 78% in the ELGAI subgroup (*p* < 0.01). The multivariate analysis did not change the results.

### MV and respiratory outcomes (Table 3)

The primary outcome, MV in the first 72 h, showed a statistically significant 28% decrease in VLGAI (p and adjusted *p* < 0.01), with a 43% decrease in the 29- to 31-week subgroup (p and adjusted *p* < 0.05), and a 23% decrease in the ELGAI subgroup (*p* < 0.05). Analyzing the primary outcome as a continuous variable showed a significant decrease in MV duration from 13 to 7 h (p and adjusted *p* < 0.05) and from 45 to 13 h (*p* < 0.01) in VLGAI and ELGAI, respectively. Significantly more infants were ventilated by HFO in both subgroups but the rate was multiplied by 4 in the 29- to 31-week stratum. The rate of administration of a second dose of surfactant significantly decreased by 62% (p and adjusted *p* < 0.01) and 67% (p and adjusted *p* < 0.01) in VLGAI and ELGAI, respectively. The INSURE practice significantly increased from 2 to 15% in VLGAI (p and adjusted *p* < 0.01) and specifically in the 29- to 31-week subgroup from 0 to 17% (*p* < 0.05) without reaching significance in the ELGAI subgroup (*p* = 0.14). In VLGAI and ELGAI, postnatal corticosteroid therapy decreased significantly by 58% (p and adjusted *p* < 0.01) and 63% (p and adjusted *p* < 0.01), respectively.

**Table 3:** Mechanical Ventilation and Respiratory Outcomes

	VLGAI		29- to 31-wk stratum		ELGAI	
	Period 1 N = 91	Period 2 N = 72	Period 1 N = 43	Period 2 N = 30	Period 1 N = 48	Period 2 N = 42
MV in the first 72 h, n (%)	72 (79)	41 (57)*##	28 (65)	11 (37)*#	44 (92)	30 (71)*
Duration of MV within the first 72 h, h (median) [1st Qu; 3rd Qu]	13 [3–48]	7*# [0–30]	8 [0–17]	0 [0–13]	45 [9–72]	13** [0–43]
Duration of MV, d (median) [1st Qu; 3rd Qu]	1 [0–6]	1 [0–6]	0 [0–1]	0 [0–1]	5 [1–24]	4 [0–10]
Duration of NIV, d (median) [1st Qu; 3rd Qu]	27 [6–40]	27 [8–35]	13 [3–28]	13 [7–25]	37 [26–45]	32 [23–39]
HFO, n (%)	26 (29)	34 (48)*##	2 (5)	6 (21)#	24 (50)	28 (67)#
2nd dose of Surfactant, n (%)	19 (21)	6 (8)*##	2 (5)	1 (3)	17 (35)	5 (12)*##
INSURE Procedure, n (%)	2 (2)	11 (15)*##	0 (0)	5 (17)*	2 (4)	6 (14)
Postnatal Corticosteroids, n (%)	28 (31)	9 (13)*##	3 (7)	1 (3)	25 (52)	8 (19)*##

*VLGAI* Very low gestational age infants < 32 weeks, *ELGAI* Extremely low gestational age infants < 29 weeks, *MV* Mechanical ventilation, *NIV* Noninvasive ventilation, *HFO* High frequency oscillation, *INSURE* Intubation-surfactant-extubation; *: *p* < 0.05; **: *p* < 0.01; #: adjusted *p* < 0.05; ##: adjusted *p* < 0.01; *#: p and adjusted *p* < 0.05; *##: p and adjusted *p* < 0.01

### Neonatal morbidity and mortality (Table 4)

BPD was significantly divided by 5 in VLGAI (p and adjusted < 0.01), disappeared in the 29- to 31-week stratum without reaching significance, and was significantly divided by 4 in the ELGAI subgroup (p and adjusted *p* < 0.05). No significant difference in overall mortality and early mortality within the first 72 h of life were shown between the two periods and subgroups. The combined BPD/mortality rate decrease of about 40% did not reach a statistically significant difference in VLGAI (*p* = 0.16) nor in the ELGAI subgroup (*p* = 0.21).

**Table 4:** Neonatal Mortality and Morbidity

	VLGAI		29- to 31-wk stratum		ELGAI	
	Period 1 N = 91	Period 2 N = 72	Period 1 N = 43	Period 2 N = 30	Period 1 N = 48	Period 2 N = 42
Physiologic BPD at 36 weeks GA, n (%)	15 (16)	2 (3) *##	4 (9)	0 (0)	11 (23)	2 (5) *#
Mortality, n (%)	10 (11)	9 (13)	1 (2)	1 (3)	9 (19)	8 (19)
Mortality within the first 72 h, n (%)	4 (4)	2 (3)	1 (2)	0 (0)	3 (6)	2 (5)
BPD and/or mortality, n (%)	25 (27)	11 (15)	5 (12)	1 (3)	20 (42)	10 (24)
Pneumothorax, n (%)	1 (1)	2 (3)	0 (0)	0 (0)	1 (2)	2 (5)
Treated PDA, n (%)	15 (17)	13 (18)	0 (0)	2 (7)	15 (31)	11 (26)
NEC ≥ grade 2a, n (%)	4 (4)	4 (6)	1 (2)	1 (3)	3 (6)	3 (7)
IVH > grade 2, n (%)	3 (3)	3 (4)	0 (0)	0 (0)	3 (6)	3 (7)
Periventricular leukomalacia, n (%)	4 (4)	0 (0)	2 (4)	0 (0)	2 (4)	0 (0)
ROP > grade 2, n (%)	1 (1)	0 (0)	0 (0)	0 (0)	1 (2)	0 (0)

*VLGAI* Very low gestational age infants < 32 weeks, *BPD* Bronchopulmonary dysplasia, *PDA* Patent ductus arteriosus, *NEC* Necrotizing enterocolitis, *IVH* Intraventricular hemorrhage, *ROP* Retinopathy of prematurity; *#: p and adjusted *p* < 0.05; *##: p and adjusted *p* < 0.01

No statistically significant difference of neonatal morbidities during both periods and subgroups was observed.

## Discussion

This retrospective cohort study compared the short-term and before discharge neonatal morbidity and mortality of a new LR strategy combining individualized increase in PEEP and SI. This LR policy is retrospectively associated with a decrease in i) endotracheal intubation and surfactant administration in the DR, ii) rate and duration of mechanical ventilation in the first 72 h, iv) administration of a 2nd dose of surfactant, v) postnatal corticosteroids treatment and vi) BPD rate. Moreover, this strategy was not associated with any significant increase in neonatal morbidity or mortality.

The adherence to the new protocol was good but could have been better as 25% of the newborns did not benefitted from the LR maneuvers. Although their baseline characteristics did not differ from the 2016 population, they were excluded from the final analysis. For the remaining infants, the not significant but expected increased median maximal SI duration and PEEP level in the lower GA-based subgroup [17], confirms the understanding by the medical team of the individualized stepwise LR maneuvers. In a hindsight, to improve the adherence, more than 6 months should have been probably dedicated to educate the medical team and/or a video recording and reviewing program implemented.

Our significant decrease in endotracheal intubation rate in the DR is in accordance with other retrospective studies [18, 19] and 3 RCTs [20–22] on SI. However, in our study as in all retrospective studies in the DR, it is difficult to ascertain whether this decrease was related to our individualized LR maneuvers or to a longer time allowed to the infants to stabilize without intubation. Nevertheless, in favor of our LR maneuvers we observed a decrease of intubation rate in the DR with all physicians, not only the “early intubators”. Nonetheless, only an RCT could add any definitive evidence on this subject. Laryngeal closure is described as the main cause of airway obstruction [23] impeding NIV in the DR in about 26% of VLGAI [24]. In our 2016 cohort, apneic airway obstructions were also observed but only 18% of our VLGAIs were intubated for this reason in the DR, that is, on average in our medical team, twice a year per physician.

Surfactant administration in the DR decreased significantly, especially in ELGAI, showing the better transition to ex-utero life after LR maneuvers. Later, the paradoxical significant lower rate of a 2nd dose of surfactant despite the decreased FiO_2_ threshold and the increased lower SpO_2_ target range may illustrate the benefits of any recruiting maneuver: when a higher FRC is recruited before surfactant administration, more alveoli benefit from it, thus decreasing the eventual need for a 2nd dose. The same explanation may apply to the INSURE procedure: a better recruitment increasing its chance of success.

Although the total MV duration did not reach a statistically significant decrease, pulmonary morbidity was reduced, as witnessed by a significantly lessened incidence of MV in the first 72 h, duration of MV within the first 72 h, surfactant and postnatal corticosteroids administrations. However, HFO ventilation because of its early-rescue introduction, and the INSURE procedure were significantly more practiced during 2016. Although only the INSURE procedure is a long established beneficial practice [25], both changes might have contributed to some pulmonary protection.

A drastic fall in BPD rate was observed in both subgroups, without reaching significance in the 29- to 31-week subgroup despite its disappearance, probably because the relatively low prevalence of BDP in this subgroup makes a significant decrease more difficult to achieve.

Our data showed a steady state in mortality and early mortality within the first 72 h of life. This is unlike the large multicentric “Sustained Aeration of Infants Lungs” (SAIL) trial comparing SI with conventional ventilation in the DR which was recently cancelled (after 426 infants analyzed) because of a significant excess of deaths at less than 48 h of age in the intervention arm without any reduction in BPD rates in preterm infants < 27 weeks GA [26]. Our speculative explanation of this harmful finding could be that the most immature infants may require an individualized and gentler support than a 15 s SI from the outset, explaining the initial persistent bradycardia observed in the SI group of the SAIL trial, and reflecting potential lung overdistention. In fact, our available data in preterm infants < 27 weeks GA show that only 27% required an SI = 15 s with a PEEP = 15 cmH2O.

The combined BPD and/or mortality rate decreased by more than 40% but without reaching significance. The paradoxical absence of lowered mortality despite the better pulmonary outcomes may be related to an outbreak of first week late-onset septic shock during 2016 (i.e., 6 septic shock cases instead of 2 during the first period) while the overall rate of culture-proven sepsis remained steady (8 then 9 cases per year). We speculate that this increased mortality by septic shock might be related to some delay in sepsis management among medical and nurse teams unaccustomed to early NIV.

Our results are in accordance with all other retrospective studies [18, 19, 27, 28] assessing SI. Only one [18] showed a decrease in BPD and severe IVH, but these results could be related to other interventions in the DR such as an individualized approach to intubation, more CPAP use, and novel thermoregulation interventions. Four RCTs showed no benefits at all [20, 29–31]. However, according to the meta-analysis of Bruschettini et al. [10], a decreased duration of MV was found (mean difference: − 5.37 days; 95% CI: − 6.31 to − 4.43), but without differences in the rate of BPD nor combined mortality/BPD. Whether our positive results could be explained only by the adjunction of the possibly more protective dynamic PEEP LR manoeuver [32] should be addressed in a specific RCT.

Two studies using a prophylactic approach with a fixed duration of SI [31, 33] have shown a nonsignificant increase in the rate of pneumothorax from 1 to 6% (OR = 4.57; 95% CI: 0.97–21.5; *p* = 0.06) [31] and from 0 to 3% (*p* = 0.08) [33]. Only one more case was found in our study, despite the application of repeated SIs associated with much higher PEEPs. To explain this discrepancy, we postulate that our individualized approach might play a fundamental role. The rate of treated PDA was slightly but significantly increased in the meta-analysis of Schmölzer et al. (RR = 1.27 (1.05–1.54)) [34], but our data do not support this observation.

A nonsignificant 27% increase in the rate of cesarean sections was observed during 2016 (*p* = 0.06). Paradoxically, our results show immediate benefits of the LR strategy, despite the absence of improved clearance of lung fluid that occurs during vaginal delivery [35]. Until recently, one of the described mechanisms of airway liquid clearance at birth was Na^+^ uptake across the airway epithelium. However, this cellular mechanism develops only in late gestation [36], and is too slow to clear the volume of liquid to be expelled within seconds to minutes after birth from airways. Therefore, the airway liquid clearance after birth is thought to result from an increase in the transepithelial pressure gradient, occurring during inspiration, which is incompletely effective in premature neonates [7]. Thus, our results might validate that personalized LR maneuvers helped to clear the fluid-filled airways and initiate gas exchange.

This study shares the limitations of monocentric retrospective cohort studies. Thus, the steady high rate of IUGR observed in our population (~ 24%) could account for a greater rate of morbidities. Given the changes that occurred in our neonatal management, confounding factors were introduced such as lowering the FiO2 threshold for surfactant administration, INSURE procedure, volume guarantee ventilation, early-rescue HFO, permissive hypercapnia, individualized doubling of the caffeine maintenance dose, and individualized sedation-analgesia. All of these factors may have protected the developing lung, and heavily influenced the pulmonary outcomes. Moreover, the multiple secondary statistical analyses made in this study limit the value of significant results. Therefore, the beneficial pulmonary outcomes must be interpreted very cautiously except probably for the intubation and surfactant administration in the DR which could not be impacted by the subsequent management changes.

## Conclusions

In conclusion, this retrospective study shows the feasibility of an individualized LR strategy based on a stepwise increase in PEEP and SI with potentially beneficial short-term neonatal outcomes. A large RCT is needed to confirm these results.

## Data Availability

The datasets used and analyzed during the current study are available from the corresponding author on reasonable request.

## Abbreviations

BPD: Bronchopulmonary dysplasia
CPAP: Continuous positive airway pressure
CPR: Cardiopulmonary resuscitation
DR: Delivery room
ELGAI: Extremely low gestational age infants
FiO2: Fraction of inspired oxygen
GA: Gestational age
HR: Heart rate
HFO: High frequency oscillation
INSURE: Intubation-surfactant-extubation
IUGR: Intrauterine restriction growth
IVH: Intraventricular hemorrhage
LR: Lung recruitment
MV: Mechanical ventilation
NEC: Necrotizing enterocolitis
NICU: Neonatal intensive care unit
NIV: Noninvasive ventilation
PDA: Patent ductus arteriosus
PEEP: Positive end-expiratory pressure
RCT: Randomized controlled trial
ROP: Retinopathy of prematurity
SI: Sustained inflation
SpO2: Pulse oxygen saturation
VLGAI: Very low gestational age infants
